# The ethics of animal research: a survey of the public and scientists in North America

**DOI:** 10.1186/s12910-016-0100-x

**Published:** 2016-03-29

**Authors:** Ari R. Joffe, Meredith Bara, Natalie Anton, Nathan Nobis

**Affiliations:** Faculty of Medicine, Department of Pediatrics, Stollery Children’s Hospital, University of Alberta, Edmonton, AB Canada; John Dossetor Health Ethics Center, University of Alberta, Edmonton, AB Canada; Faculty of Medicine, University of Alberta, Edmonton, AB Canada; Morehouse College, Department of Philosophy, Atlanta, USA; 4-546 Edmonton Clinic Health Academy, 11405 87 Avenue, Edmonton, T6G 1C9 AB Canada

**Keywords:** Animal models, Animal research, Ethics, Methodology

## Abstract

**Background:**

To determine whether the public and scientists consider common arguments (and counterarguments) in support (or not) of animal research (AR) convincing.

**Methods:**

After validation, the survey was sent to samples of public (Sampling Survey International (SSI; Canadian), Amazon Mechanical Turk (AMT; US), a Canadian city festival and children’s hospital), medical students (two second-year classes), and scientists (corresponding authors, and academic pediatricians). We presented questions about common arguments (with their counterarguments) to justify the moral permissibility (or not) of AR. Responses were compared using Chi-square with Bonferonni correction.

**Results:**

There were 1220 public [SSI, *n* = 586; AMT, *n* = 439; Festival, *n* = 195; Hospital *n* = 107], 194/331 (59 %) medical student, and 19/319 (6 %) scientist [too few to report] responses. Most public respondents were <45 years (65 %), had some College/University education (83 %), and had never done AR (92 %). Most public and medical student respondents considered ‘benefits arguments’ sufficient to justify AR; however, most acknowledged that counterarguments suggesting alternative research methods may be available, or that it is unclear why the same ‘benefits arguments’ do not apply to using humans in research, significantly weakened ‘benefits arguments’. Almost all were not convinced of the moral permissibility of AR by ‘characteristics of non-human-animals arguments’, including that non-human-animals are not sentient, or are property. Most were not convinced of the moral permissibility of AR by ‘human exceptionalism’ arguments, including that humans have more advanced mental abilities, are of a special ‘kind’, can enter social contracts, or face a ‘lifeboat situation’. Counterarguments explained much of this, including that not all humans have these more advanced abilities [‘argument from species overlap’], and that the notion of ‘kind’ is arbitrary [e.g., why are we not of the ‘kind’ ‘sentient-animal’ or ‘subject-of-a-life’?]. Medical students were more supportive (80 %) of AR at the end of the survey (*p* < 0.05).

**Conclusions:**

Responses suggest that support for AR may not be based on cogent philosophical rationales, and more open debate is warranted.

**Electronic supplementary material:**

The online version of this article (doi:10.1186/s12910-016-0100-x) contains supplementary material, which is available to authorized users.

## Background

Given the massive public funding of animal research (AR) in democratic societies, it might be expected that the arguments for and against AR are well settled [1, 2]. However, the details of standard ethical arguments and counterarguments for AR are not often publically discussed, and it is likely that most people are not aware of the details of the debate. This is also true in the peer-reviewed medical literature, where open debate including details of all the arguments is uncommon. Nevertheless, AR is an ethical issue because animals are harmed in experimentation from such things as confinement, fear, pain, and early death [3, 4]. In this study, we refer to AR which is harmful (detrimental to some significant interest the being has, such as the interest in maintaining life and bodily integrity, and avoiding pain and frustration), non-therapeutic (does not aim at restoring the health of a research subject with prior injury/disease), and non-consensual (conducted with subjects who have not voluntarily agreed to participate), and thus would be unethical if done in a non-research setting [4].

The question to debate is this: is any or all AR that involves seriously harming animals morally permissible? The moral justification is usually given by one of three types of arguments [5–7]. “Benefits arguments” claim that AR benefits humans greatly, is necessary for human benefit, or that there are no alternatives for human benefit; these are the most common justifications given by pro-AR groups [8–10]. “Characteristics of non-human-animals (NHA) arguments” claim that animals are property, are not sentient, or that animals harm other animals; these arguments led to the initial development of AR and its legal regulation [11]. “Human exceptionalism arguments” claim that humans have more advanced abilities, are of a special ‘kind’, can enter into contracts, or must sacrifice NHAs in their lifeboat [12–15]. Importantly, the first two types of arguments actually rely on human exceptionalism arguments: to justify using animals [as necessary] for human benefits, or as property, requires an argument for why humans cannot be used in the same way [12]. Previous surveys have generally asked only whether people support AR for human benefit, and have not asked people to evaluate their reasons for supporting (or not) AR [16–20]. When we presented common arguments and counterarguments to a small sample of pediatric health care workers at our children’s hospital, we found that most were not convinced of the moral permissibility of AR [21]. Here, we survey a large sample of the public, medical students, academic pediatricians, and scientists to determine their considered opinions regarding the moral permissibility of AR. We aimed to determine not simply whether the public, medical students, academic pediatricians, and scientists support AR, but whether they think the usual arguments (and counterarguments) in support (or not) of AR are convincing. Our objective in this exploratory research was to determine how strong commitment was to each argument in favor of animal research, in the face of the counterarguments presented.

## Methods

### Questionnaire development

The development and reporting of the questionnaire followed published recommendations [22]. As described elsewhere, this included a literature search, content and construct validation by non-author experts using a clinical sensibility tool and tables of specifications (*n* = 4 experts), face and content validation by pilot testing using a clinical sensibility tool and informal semi-structured interviews (to ensure clarity, realism, validity, and ease of completion) (*n* = 14 individuals) [21]. Not all of the authors, or the experts and public validators were in favor of AR. The study was approved by the health research ethics board of the University of Alberta (Pro00039590), and return of the survey was considered consent to participate.

### Questionnaire administration

We surveyed the public (4 groups), adults with biomedical science training (2 groups), and animal researchers (2 groups), the details of which are as follows:

1. Public: we chose 4 groups that represented a range of situations that were conveniently accessible for survey distribution: i) A convenience sample of adults approached while in various line-ups at the Heritage Festival in Edmonton, Alberta, Canada in August 2013 (*n* = 195). These adults were asked if they would fill out a paper survey about AR in return for $5 food ticket as an incentive. We did not track the number of people asked to participate, but our impression was that most adults approached completed the survey. ii) A random sample of Canadian adults accessed using the marketing firm Survey Sampling International (SSI) in November 2014 (*n* = 586). The sample “was selected to be reflective of the Canadian population over age 18 years with at least a high school education, by age, gender, race/ethnicity, income, and geographic region.” Of those invited to participate, 1 terminated, 85 submitted partial responses, and 501 submitted complete responses. iii) A sample of US adults using Amazon Mechanical Turk (AMT) in February 2015 (*n* = 439). For this sample, we limited potential responders to those living in the US, with a Human Intelligence Task approval rate >90 %, and we paid $1.60 on completion. The survey was on the AMT site for <4.5 h, and the average time spent per survey was 31 min. Crowdsourcing on AMT has found results to be psychometrically valid, with high test-retest reliability, attentiveness, and truthfulness, even on complex cognitive and decision-making tasks [23–27]. We included two attention checks with excellent results (one-third of the way in: “please tell us whether you agree with this equation: 2 + 2 = 4”; 407/18 (97 %) agree; and toward the end of the survey: “to show you have read the instructions, please check ‘none of these’ as your answer to this question”; 413/415 (99.5 %) ‘none of these’). iv) A convenience sample of adult visitors on the pediatric wards of the Stollery Children’s Hospital, Edmonton, Alberta, Canada in May 2015 (*n* = 107). We did not track the number of people asked to participate, but our impression was that most of those approached completed the paper survey.

2. Adults with biomedical science training: i) The second year University of Alberta Medical School in September 2013, and ii) The next second year class in November 2014. Non-respondents were sent three reminders at about 2–3 week intervals.

3. Animal researchers: i) The corresponding authors of AR papers published during the 6 months from October 2012 to March 2013 in the high impact journals Nature, Science, and Critical Care Medicine (i.e., representing leaders in their field of AR); and ii) All academic faculty pediatricians at The Hospital for Sick Children in Toronto, Ontario, Canada, as listed by email on the University of Toronto website. We assumed these pediatricians would have many ties to research activities, including knowledge of, if not participation in, AR. Non-responders were sent three reminders at about 2–3 week intervals.

The surveys were done using the web-based tool REDCap, which allows anonymous survey responses to be collected or entered, and later downloaded into statistical software for analysis [28]. The paper surveys done at the local festival and the children’s hospital were entered by hand, whereas all other groups entered data directly into the REDCap surveys after invitation by email or using the SSI and AMT platforms.

### Questionnaire content

The background section stated “In this survey, ‘animals’ means: mammals, such as mice, rats, dogs, and cats. It has been estimated that over 100 million animals are used in the world for research each year. There are many good reasons to justify animal research, which is the topic of this survey. Nevertheless, some people argue that these animals are harmed in experimentation, because their welfare is worsened. In this survey, ‘harmful’ means such things as: pain, suffering (disease/injury, boredom, fear, confinement), and early death. We value your opinion on the very important issue of the ethical dimension of animal research.” We chose mammals to represent a group of sentient animals that are thus capable of being harmed.

We presented demographic questions, 3 questions about support for AR, and 12 arguments with their counterarguments to consider. The survey stated: “a) First, we give you an argument to justify harmful animal research, and we ask if you agree with that argument; b) Then we give some responses to the argument, and we ask if you think each response would make it harder for someone to justify harmful animal research using the initial argument (i.e., would make the initial argument less convincing). All the arguments and responses in this survey are those commonly made in the literature on animal research.” When each argument was presented, it was followed with the question “Is this a good enough reason to justify using animals in medical research?” When the responses to an argument were presented, they were followed with the question: “Do any of the following responses make it harder for someone to justify animal research using Argument x (i.e., make Argument x much less convincing)?”

### Statistics

We used REDCap as our web-based survey management tool, allowing anonymous survey responses to be collected, and later downloaded into an SPSS database for analysis [28]. Responses are described using proportions (percentages). Pre-specified subgroup analyses included exploratory comparisons on all responses for: the 4 public groups; the two medical school classes; and the two animal researcher groups. If results were not statistically and clinically significantly different within each of these groups, we planned to compare the groups to each-other. These comparisons were done using the Chi-square statistic, with *P* < 0.05 after Bonferroni correction for multiple comparisons considered statistically significant. Prior to statistical analysis, we defined a clinically significant difference between groups as one where the comparison is statistically significant, in addition to having a clear majority (at least 60 %) on different sides of the yes/no response option. This was done because we are interested in whether the groups have opinions that could result in different practical consequences both for the animals, and for the researchers involved. For example, if many respondents have very different opinions about the moral permissibility of AR, this might lead to very different levels of support of AR, and thus have different implications for the actual practice of AR.

## Results

### Public responses

#### Demographics

There were 195 respondents from the local festival, 586 from SSI, 439 from AMT, and 107 from the children’s hospital samples. Most respondents were under 45 years old, had at least some college or university training, and the vast majority had never done AR (in Additional file 1: Table E-1).

#### Benefits arguments and counterarguments

Responses are shown in Table 1, and (see Additional file 1: Table E-2). About half of respondents accepted the benefits arguments that “AR benefits humans greatly” (55 %), “Animal experimentation is necessary for human benefit” (50 %); and “There are no alternatives to animal experimentation” (41 %) as good enough to justify AR. Far fewer accepted the argument that “Humans naturally need to seek knowledge” (24 %). Many found the counterarguments convincing, including those who initially responded that the argument was enough to justify AR. Most were convinced by counterarguments suggesting that there are alternative experimental methods (84 %), or that more effort must be devoted to developing alternative methods (79 %). About half found counterarguments convincing that pointed out that if AR [is necessary] for great benefits to humans, this should also justify using humans in the same experiments. There were few statistically significant differences among the public samples, with none being clinically significant.

**Table 1:** Results for questions about “Benefits Arguments” to morally justify animal research

Argument (A)/Counterargument (CA)						
	Group	Is this a good enough reason to justify using animals in medical research?		Do any of the following responses make the argument much less convincing?		Of those convinced: proportion who judged the counterargument as persuasive
		Yes	No	Yes	No	
A1. Animal experimentation benefits humans greatly.^a^						
	Public	698/1270 (55 %)	572/1270 (45 %)			
	Med School	140/188 (74 %)	48/188 (26 %)			
CA: If great human benefits justify using animals in medical research, this should also justify using humans in the same medical research.^a^						
	Public			610/1260 (48 %)	650/1260 (52 %)	291/692 (42 %)
	Med School			48/188 (26 %)	140/188 (74 %)	25/140 (18 %)
CA: If animals can experience pain and suffering, it remains unclear why we morally may use them in experiments for human benefit.^a^						
	Public			792/1264 (63 %)	472/1264 (37 %)	348/691 (50 %)
	Med School			95/188 (51 %)	93/188 (49 %)	60/70 (86 %)
A2: Animal experimentation is necessary for human benefit.^a^						
	Public	621/1246 (50 %)	625/1246 (50 %)			
	Med School	126/181 (70 %)	55/181 (30 %)			
CA: More humans would benefit if the money spent on animal experiments was instead devoted to humanitarian aid (for example, in developing countries).						
	Public			584/1249 (47 %)	665/1249 (53 %)	226/613 (37 %)
	Med School			67/180 (37 %)	113/180 (63 %)	42/126 (33 %)
CA: There are now alternative experimental methods that do not use animals and that allow science to advance.^a^						
	Public			1049/1244 (84 %)	195/1244 (16 %)	482/612 (79 %)
	Med School			130/181 (72 %)	51/181 (28 %)	87/126 (69 %)
CA: It is unclear why the statement 'animal experimentation is necessary for human benefits' justifies animal experiments, but the statement 'human experimentation is necessary for human benefits' does not justify the same experiments on humans.^a^						
	Public			667/1238 (54 %)	571/1238 (46 %)	245/612 (40 %)
	Med School			62/180 (34 %)	118/180 (66 %)	32/126 (25 %)
A3: There are no alternatives to animal experimentation.^a^						
	Public	507/1240 (41 %)	733/1240 (59 %)			
	Med School	98/172 (57 %)	74/172 (43 %)			
CA: Researchers have not looked hard enough for alternatives to animal experimentation. For example, since using animals to test drugs has been required by law, researchers may have assumed that there is no other way.^a^						
	Public			801/1235 (65 %)	434/1235 (35 %)	280/498 (56 %)
	Med School			81/171 (47 %)	90/171 (53 %)	38/96 (40 %)
CA: If more effort was devoted to developing alternative research methods that do not use animals, animal experimentation may not be necessary anymore.^a^						
	Public			985/1239 (79 %)	254/1239 (21 %)	352/501 (70 %)
	Med School			106/171 (62 %)	65/171 (38 %)	55/96 (57 %)
A4: Humans naturally need to seek knowledge.^a^						
	Public	293/1240 (24 %)	947/1240 (76 %)			
	Med School	19/169 (11 %)	150/169 (89 %)			
CA: This can justify almost anything, including harmful experiments on humans against their will, in order to gain knowledge.^a^						
	Public			690/1227 (56 %)	537/1227 (44 %)	127/289 (44 %)
	Med School			126/168 (75 %)	42/168 (25 %)	5/19 (26 %)
CA: We have learned a great deal from earthquakes, fires and warfare; but, this does not justify recreating these things in order to gain more knowledge.						
	Public			859/1231 (70 %)	371/1231 (30 %)	167/287 (58 %)
	Med School			121/168 (72 %)	47/168 (28 %)	10/19 (53 %)

^a^Statistically significant difference between public and medical students (*p* < 0.05 after Bonferroni correction). Clinically significant difference between public and medical students (statistically significant, and a clear majority of at least 60 % on opposite sides of the yes/no response option): none

#### Characteristics of NHA arguments and counterarguments

Responses are shown in Table 2 and (see Additional file 1: Table E-3). Almost all respondents were not convinced by these arguments, and each counterargument explained this for about half of respondents. The statistically significant differences in some responses were only clinically significant for one (more respondents on AMT were convinced by the counterargument that “this would mean that a pet cat or dog is simply a living machine, without any feelings”).

**Table 2:** Results for questions about “Characteristics of non-human-animals arguments” to morally justify animal research

Argument (A)/Counterargument (CA)						
	Group	Is this a good enough reason to justify using animals in medical research?		Do any of the following responses make the argument much less convincing?		Of those convinced: proportion who judged the counterargument as persuasive.
		Yes	No	Yes	No	
A1. Animals harm other animals.						
	Public	153/1237 (12 %)	1084/1237 (88 %)			
	Med School	7/169 (4 %)	162/169 (96 %)			
CA: It is unclear why we should take this (we may harm animals) as moral advice from animals, but not take other moral advice from animals (for example, animals rape and kill members of their own species would mean we may rape and kill humans). In other words, animals are not qualified to give moral advice.^a^						
	Public			655/1227 (53 %)	572/1227 (47 %)	80/148 (54 %)
	Med School			114/168 (68 %)	54/168 (32 %)	2/7 (29 %)
A2: Animals cannot really feel anything. They are simply living machines.						
	Public	84/1239 (7 %)	1155/1239 (93 %)			
	Med School	9/168 (5 %)	159/168 (95 %)			
CA: This would mean that a pet cat or dog is simply a living machine, without any feelings like happiness, sadness, fear or pain.^a^						
	Public			574/1237 (46 %)	663/1237 (54 %)	60/83 (72 %)
	Med School			118/167 (71 %)	49/167 (29 %)	6/9 (67 %)
A3: Animals are property.						
	Public	179/1215 (15 %)	1036/1215 (85 %)			
	Med School	9/161 (6 %)	152/161 (94 %)			
CA: Since animals can desire things, intentionally act to fulfill those desires, and can understand (even dimly) that it is me that wants something and is trying to get it, they are not simply property.						
	Public			759/1212 (63 %)	453/1212 (37 %)	84/176 (48 %)
	Med School			110/160 (69 %)	50/160 (31 %)	2/9 (33 %)

^a^Statistically significant difference between public and medical students (*p* < 0.05 after Bonferroni correction). Clinically significant difference between public and medical students (statistically significant, and a clear majority of at least 60 % on opposite sides of the yes/no response option): none

#### Human exceptionalism arguments and counterarguments

Responses are shown in Table 3 and (see Additional file 1: Table E-4). Only a minority of respondents accepted these arguments as good enough to justify AR (18–36 %). For most respondents, the stated counterarguments explained this lack of acceptance of the initial arguments. For example, the counter-arguments from species overlap [“not all humans have these abilities”], that animals are “subjects of a life” [“of the ‘kind’ able to have experiences, memories, and preferences”], and that similar arguments of prejudice were used in the past [“it is unclear why caring more about someone justifies harming those we care less about. For example, in the past this argument was used to justify prejudice (for example, slavery) against those we cared less about, who were considered not of our own kind”] were convincing for most (60 %, 55 %, and 58 % respectively). The few statistically significant differences between the public groups were clinically significant for one (more respondents on AMT were convinced by the counterargument that “this would mean we have no direct moral duties to humans who cannot enter into this contract. For example, babies, and severely brain-damaged people”).

**Table 3:** Results for questions about “Human exceptionalism” arguments to morally justify animal research

Argument (A)/Counterargument (CA)						
	Group	Is this a good enough reason to justify using animals in medical research?		Do any of the following responses make the argument much less convincing?		Of those convinced: proportion who judged the counterargument as persuasive.
		Yes	No	Yes	No	
A1.Humans have more advanced mental abilities than animals, like knowing right from wrong, having empathy, planning for the future, and being able to read and talk.						
	Public	296/1235 (24 %)	939/1235 (76 %)			
	Med School	46/166 (28 %)	120/166 (72 %)			
CA: Not all humans have these abilities. Babies, infants, and severely brain damaged children or adults (for example, with very advanced Alzheimer’s) do not have these abilities. Some animals may have more abilities than these humans.						
	Public			732/1226 (60 %)	494/1226 (40 %)	142/293 (48 %)
	Med School			101/165 (61 %)	64/165 (39 %)	17/46 (37 %)
CA: This means having superior abilities [humans] justifies actively harming those with inferior abilities [animals]. It is unclear why, if animals can experience pain and suffering, having lower mental abilities makes it acceptable to use them in experiments. For example, sometimes humans with superior abilities [adults] have many obligations to those with inferior abilities [children].^a^						
	Public			634/1217 (52 %)	583/1217 (48 %)	147/289 (51 %)
	Med School			119/166 (72 %)	47/166 (28 %)	21/46 (46 %)
A2: Humans are a special kind or group. We care more about this kind, and have more obligations to this kind.^a^						
	Public	351/1223 (29 %)	872/1223 (71 %)			
	Med School	71/165 (43 %)	94/165 (57 %)			
CA: Imagine there is a more advanced species than humans. This would mean that they are justified in using humans in experiments, because they care more about their own kind.						
	Public			634/1213 (52 %)	579/1213 (48 %)	180/345 (52 %)
	Med School			96/164 (59 %)	68/164 (41 %)	28/71 (39 %)
CA: Maybe humans are of the kind ‘able to experience suffering and pleasure’ (sentient being). If so, our kind includes animals.						
	Public			649/1217 (53 %)	568/1217 (47 %)	138/349 (40 %)
	Med School			80/163 (49 %)	83/163 (51 %)	28/71 (39 %)
CA: Maybe humans are of the kind ‘able to have experiences, memories, and preferences’ (subject of a life). If so, our kind includes animals.						
	Public			673/1213 (55 %)	540/1213 (45 %)	141/348 (41 %)
	Med School			84/163 (52 %)	79/163 (48 %)	27/71 (38 %)
CA: It is unclear why caring more about someone justifies harming those we care less about. For example, in the past this argument was used to justify prejudice (for example, slavery) against those we cared less about, who were considered not of our own kind.^a^						
	Public			700/1208 (58 %)	508/1208 (42 %)	176/346 (51 %)
	Med School			120/164 (73 %)	44/164 (27 %)	45/71 (63 %)
A3: We have moral duties only to those who can agree to the same duties. This is like a contract between people in society. Since animals cannot enter into this contract with humans, we do not have moral duties to animals.						
	Public	218/1223 (18 %)	1005/1223 (82 %)			
	Med School	25/164 (15 %)	139/164 (85 %)			
CA: This would mean we have no direct moral duties to humans who cannot enter into this contract. For example, babies, and severely brain-damaged people.^a^						
	Public			577/1216 (47 %)	589/1216 (48 %)	94/215 (44 %)
	Med School			109/163 (67 %)	54/163 (33 %)	7/25 (28 %)
A4: Evolution, and our nature, dictates that we must make sure we survive as a species.						
	Public	418/1214 (34 %)	796/1214 (66 %)			
	Med School	57/162 (35 %)	105/162 (65 %)			
CA: It is unclear why what we evolved to do [survive at all costs] is what we morally should do. In other words, evolution does not take moral considerations into account.						
	Public			666/1208 (55 %)	542/1208 (45 %)	201/412 (49 %)
	Med School			94/163 (58 %)	69/163 (42 %)	20/57 (35 %)
CA: Research is unlikely to save our species; it is for the benefit of some humans, not the whole species (which is what evolution is about).						
	Public			550/1203 (46 %)	653/1203 (54 %)	153/410 (37 %)
	Med School			56/163 (34 %)	107/163 (66 %)	9/57 (16 %)
A5: We must sacrifice one (animals) in order to save another (humans). This is like being in a lifeboat on the ocean where we must throw one overboard or the lifeboat will sink.						
	Public	438/1212 (36 %)	774/1212 (64 %)			
	Med School	74/163 (45 %)	89/163 (55 %)			
CA: Most people would throw a dog overboard to save humans in the lifeboat; but, this does not mean that the dog can be used in experiments. For example, some might throw an elderly man overboard to save their children in the lifeboat; but, this does not mean elderly men can be used for experiments.						
	Public			650/1206 (54 %)	556/1206 (46 %)	179/435 (41 %)
	Med School			75/163 (46 %)	88/163 (54 %)	23/74 (31 %)

^a^Statistically significant difference between public and medical students (*p* < 0.05 after Bonferroni correction). Clinically significant difference between public and medical students (statistically significant, and a clear majority of at least 60 % on opposite sides of the yes/no response option): none

#### General questions

Reponses are shown in Table 4 and (see Additional file 1: Table E-5). At the beginning and again at the end of the survey we asked if “in order to achieve human benefits, research that results in harm to animals should be supported”; 44 % and 41 % responded “yes” respectively. Finally, when asked “what is it about vulnerable humans that makes it wrong to use them in experiments”, the most common response was “they are still human” (49 %). The responses to these questions were statistically significantly different, although not clinically significant.

**Table 4:** Responses to general questions about support for animal research

Question	Group	Yes	No	I have never thought about whether AR should be supported
In order to achieve human benefits, research that results in harm to animals (such as pain, suffering and early death) should be supported.^a^	Public	569/1303 (44 %)	550/1303 (42 %)	184/1303 (14 %)
	Medical School	168/209 (80 %)	23/209 (11 %)	18/209 (9 %)
Considering all the arguments and responses in this survey, we want to ask you again.^a,b^	Public	502/1213 (41 %)	711/1213 (60 %)	-
	Medical School	128/161 (80 %)	33/161 (20 %)	-
Of those who originally said “yes” or “have never thought about whether to support AR”.	Public	-	229/692 (33 %)	-
	Medical School	-	22/148 (15 %)	-
What is it about vulnerable humans (for example babies, severely brain damaged people, people with very advanced Alzheimers) that makes it wrong to use them in experiments?^a^	These vulnerable humans are able to experience things like pleasure, joy, happiness, sadness, pain, and suffering	These humans are vulnerable to physical and psychological harm; using them in experiments is harmful for them	We care about them	They are still human
Public	268/1188 (23 %)	208/1188 (18 %)	125/1188 (11 %)	587/1188 (49 %)
Medical School	18/161 (11 %)	33/161 (20 %)	14/161 (9 %)	96/161 (60 %)

^a^Statistically significant difference between public and medical students (*p* < 0.05 after Bonferroni correction). ^b^Clinically significant difference between public and medical students (statistically significant, and a clear majority of at least 60 % on opposite sides of the yes/no response option)

### Adults with biomedical science training (Medical School Classes)

#### Demographics

The response rate was 112/164 (68 %) and 82/167 (49 %) in the two medical school classes. Most respondents were under 35 years, and 60–62 % had never done AR (in Additional file 1: Table E-1). There were no statistically significant differences between the two medical classes in the responses to any question, and results from both are presented together below. Given only 2 clinically significant differences among the public survey groups for responses to arguments, we pooled the public responses for comparison to medical students.

#### Benefits arguments and counterarguments

Responses are shown in Table 2. Similar to the public, most respondents accepted the ‘benefits arguments’ and were convinced by counterarguments suggesting that there are alternative experimental methods. Eleven of these 13 questions had statistically significant differences between the public and medical student responses; although not meeting our clinically significant criterion, consistently, medical students were more convinced by benefits arguments, and less convinced by their counterarguments compared to the public. For example, only a minority found counterarguments convincing that pointed out that if AR [is necessary] for great benefits to humans, this should also justify using humans in the same experiments (26–34 %).

#### Characteristics of NHA arguments and counterarguments

Responses are shown in Table 3. Almost all respondents were not convinced by these arguments, and the counterarguments explained this for most of respondents. There were two statistically significant differences from the public responses, but these were not clinically significant.

#### Human exceptionalism arguments and counterarguments

Responses are shown in Table 4. Similar to the public, only a minority of respondents accepted these arguments as good enough to justify AR, and the stated counterarguments explained this lack of acceptance of the initial arguments. The four statistically significant differences between the public and medical students were not clinically significant; however, consistently, the medical students were more convinced by several of the counterarguments.

#### General questions

Responses are shown in Table 4. When asked at the beginning and again at the end of the survey if “in order to achieve human benefits, research that results in harm to animals should be supported”; 80 % and 80 % responded “yes” respectively. Compared to the public, this high level of support for AR was statistically and clinically significant. Finally, when asked “what is it about vulnerable humans that makes it wrong to use them in experiments”, the most common response was “they are still human” (60 %), statistically more often than for the public.

### Animal researchers

Corresponding authors (*n* = 178) were invited to participate by e-mail. The response rate was 5/178 (3 %) after 4 mailings; therefore, no useful results can be reported here. Academic pediatricians at the Hospital for Sick Children (*n* = 141) were invited to participate by e-mail. The response rate to the demographics questions was 18/141 (13 %); however, before all the benefits arguments were presented the response rate dropped to only 14/141 (10 %), and therefore no useful results can be reported here. The difference in response rates between medical students and animal researchers was statistically significant (Chi-square *p* < 0.001).

## Discussion

There are several important findings from this survey. First, the public (44 %) and medical students (80 %) are supportive of AR. Second, ‘benefits arguments’ were usually thought sufficient to justify AR. However, when confronted with counterarguments pointing out that alternative research methods may be available, most were not as convinced of the initial argument. In addition, counterarguments suggesting that it is unclear why the same ‘benefits arguments’ do not apply to using humans in the same medical experiments were convincing for about half of the public and one third of medical students. Third, almost all respondents were not convinced by “characteristics of NHA arguments”, including that NHA may not be sentient or are simply property. Fourth, most respondents were not convinced by ‘human exceptionalism’ arguments; these are the arguments that justify AR due to benefits but claim the same benefits do not justify human research. These include arguments that humans have more advanced mental abilities than NHAs, are of a special ‘kind’, can enter into social contracts, or face a lifeboat situation where human interests trump NHA interests. Fifth, common counterarguments explained much of the respondents’ lack of acceptance of ‘human exceptionalism’ arguments: the ‘argument from species overlap’ (pointing out that not all humans have these more advanced abilities, while some animals do) [29–31]; the notion that ‘kind’ is vague (why are we not of the kind ‘sentient animal’ or ‘subject-of-a-life’?) [32]; and the notion of ‘kind’ has been used in the past to justify prejudice against those society cared less about [33]. Sixth, there were several differences between the public and medical school student responses; however, most were not clinically significant, although medical students seemed consistently more convinced by benefits arguments and less convinced by their counterarguments. Medical students were much more supportive of AR, even at the end of the survey (80 %). Finally, animal researchers did not agree to engage with this survey, with too few responses to report any data.

Previous surveys of the public and scientists have generally asked only whether people support AR for human benefit, and not asked people to evaluate their reasons for supporting (or not) AR (Table 5) [16–20, 34]. The few surveys that have asked for some elaboration on reasons for supporting AR did not ask for the amount of detail, or explore response to counterarguments, as in our survey (Table 5) [35, 36]. These prior surveys do suggest that people believe there are no alternatives to AR to achieve great human benefits, and that the moral justification for using NHA and not humans is not clear. Our survey supports and expands on these prior findings, with several suggested implications. First, the findings for ‘benefits arguments’ and ‘human exceptionalism’ arguments and their counterarguments suggest that public and medical student support for AR may not be based on cogent philosophical rationales. We suggest that the support may be based on group membership effects, with commitment to a current ‘Kuhnian’ scientific research paradigm of AR, without knowledge of or serious engagement with the detailed arguments [37]. Similarly, the Oxford Animal Ethics Center has called this institutionalization of the practice of AR “normalizing the unthinkable.” [38] Second, the findings for the ‘characteristics of NHA arguments’ and their counterarguments suggest that current AR animal protection practices may not be in line with the public and medical student beliefs about NHAs. For example, legal protections of NHAs are based on the assertion that these NHAs are property [39], and belief that NHAs are sentient is the basis for the counterarguments that question the moral permissibility of AR (e.g., argument from species overlap). Third, counterarguments suggesting that “researchers have not looked hard enough for alternatives to animal experimentation” and “if more effort was devoted to developing alternative research methods that do not use animals, animal experimentation may not be necessary anymore” were convincing for most respondents. Thus, a focus on return on investment from AR and alternative research methods may help people in considering the ethics of AR [2, 40, 41]. The translation rate into human benefit (i.e., accuracy of research models) is very low for AR, on the order of 0–8 % in all fields it has been examined in, far short of reliably providing ‘great human benefit’ [8, 42–48]. Fourth, that animal researchers are reluctant to engage in this survey, described as an academic study from the University of Alberta and approved by our health research ethics board, is disappointing. More open discussion regarding the ethics of AR is surely warranted. Finally, that many were supportive of AR even after considering all the arguments/counterarguments, particularly for medical students, suggests that social science research is needed to determine why philosophical argumentation does not translate into practical behavior change.

**Table 5:** Recent surveys that shed light on public opinion regarding animal research

Survey	Type of question(s)	Description of main ethics findings
Eurobarometer, 2010 [16]	“Scientists should be allowed to experiment on animals like dogs and monkeys if this can help sort out human health problems?”	44 % agree; 37 % disagree
UK, Ipsos MORI, 2012 [17]	Most people (85 %) are “conditional acceptors” of AR, “so long as it is for medical research purposes” or “for life-threatening diseases” or “where there is not an alternative”, considering AR as a “necessary evil” for human benefit.	37 % were objectors (including 53 % of those age 15–24 years): they responded that they “do not support the use of animals in any experiments because of the importance I place on animal welfare” or “the government should ban all experiments on animals for any form of research.” Most (76 %) agree that “there needs to be more research into alternatives to animal experiments.”
Gallup’s Values and Beliefs survey, USA, 2011 [18]	Asked whether medical testing on animals is ‘morally acceptable’ or ‘morally wrong’.	43 % (including 54 % of those age 18–29 years) responded “morally wrong”
Gallup’s Values and Beliefs survey, USA, 2015 [19]	“Animals deserve the exact same rights as people to be free from harm and exploitation.”	32 % chose this option. When asked, “in general, how concerned are you about the way animals used in research are currently treated in the US today”, 67 % responded very or somewhat concerned.
PEW Research Center survey, USA, 2015 [20]	“All in all, do you favor or oppose the use of animals in scientific research?”	50 % oppose (up from 43 % in 2009; 62 % of women), 47 % favor, 3 % don’t know.
National Nanos RDD Crowdsource random survey of 1000 Canadians, 2013 [34]	“Would you say that the potential suffering of animals used in the following types of situations is acceptable, somewhat acceptable, somewhat unacceptable, or unacceptable?” “Some people think that the benefits of using animals to advance science and medicine outweigh the welfare of the animals while others believe that the welfare of the animal is important in determining what is an acceptable or unacceptable use of animals for science and medical testing. Which of these two views, if either best reflects your opinion?”	Response of acceptable or somewhat acceptable: testing to ensure safety and impact of medicine and medical devices, 57 %; developing products or devices for humans or animals such as artificial organs, 61 %; conducting medical research that relates to human or animal diseases or disorders, 64 %. Reponses: the benefits of using animals to advance science and medicine outweigh the welfare of the animals, 30 %; the welfare of the animal is important in determining what is an acceptable or unacceptable use of animals, 54 %.
Sweden: rheumatoid arthritis patients and scientific expert members of research ethics boards, 2014 [35]	Most respondents agreed to AR for at least some type of biomedical research. “In some research animals are used instead of people. What do you believe could be a relevant reason to expose animals to research that we ourselves would not take part in?”	Only a minority chose response options of “humans have higher moral status”, “humans have higher intelligence”, “animals do not have a soul”, or “animals suffer less than humans do.” Most chose either “there are no relevant differences” (69 % of patients and 36 % of scientists), or “there are other relevant differences” (12 % of patients and 44 % of scientists).
UK: scientists promoting animal research, lay public, and animal welfarists, 2009 [36]	The support for AR on a Likert scale of 7 was: 5.33 (SD 1.46), 3.57 (SD 1.70), and 1.48 (SD 0.87) for scientists, lay public, and animal welfarists respectively.	The differences were largely explained by the scientists’ higher perception of lack of alternatives and of humans as superior, and lower perception of animal sentience.

This study has several limitations. Response rates for medical school classes were 68 % and 49 %; thus we cannot rule out biased participation in the survey. In addition, we do not know the true response rate for our public festival, hospital wards, and AMT samples. Nevertheless, we estimate that most people approached for the paper surveys agreed to participate, the AMT surveys were completed in a very short period of time (<4.5 h on-line), and the SSI surveys were fully completed by 501/587 of those invited (85 %). Arguments and counterarguments presented needed to be short and concise, and this may have left out important details that would have influenced the understanding and response to the text. We did not ask about specific subtypes of AR and thus do not know if opinions will vary by whether research is on toxicology or efficacy, cancer or sepsis, etc. Nevertheless, the questions were framed assuming great benefit to humans from AR, and thus we believe the opinions are reflective of beliefs for types of AR that have such great benefit. We did not involve animal researchers in the development of the survey, and this may have biased the wording of the questions. Finally, our description of AR as harmful may have biased the responses. However, the survey was meant to apply to AR that is harmful to the animals; AR is a moral issue precisely because it is harmful to the animals; and, that AR is said by advocates to be done as ‘humanely’ as possible, with suffering minimized, entails that it is harmful to the animals.

Strengths of this study include the rigorous survey development process (including review by moral philosophers), and the inclusion of the most common and accepted arguments/counterarguments in the literature. This is the first survey we are aware of that asks the public and medical students to consider not just whether they support AR, but rather to consider the most common arguments in the literature in favor of and against AR. Another strength is the large sample size of the public, obtained from 4 different samples, with strikingly similar responses, enhancing the generalizability of our results. The similarity of findings to our previous sample of pediatric health care workers also enhances the generalizability of the results [21].

## Conclusion

When presented with common arguments to justify AR, most respondents accepted ‘benefits’ arguments, and only a minority found the ‘characteristics of NHA arguments’ and ‘human exceptionalism’ arguments convincing. Most found the argument to justify AR significantly weakened by common counterarguments, including those who initially found the ‘benefits’ arguments convincing. These responses to all the common arguments/counterarguments on offer in the literature regarding the moral permissibility of AR suggest that the public may want to re-consider whether public funds ought to support AR. Engagement with and serious discussion of the arguments on both sides of the AR debate by the public and scientists (including animal researchers); and deliberate extensive investigation of alternative research methods, and the return on investment from using them (as compared to the poor track record of AR [8, 38, 42–48]) are potential ways forward in the debate about the moral permissibility of AR.

### Ethics approval and consent to participate

The study was approved by the health research ethics board of the University of Alberta (Pro00039590), and return of the survey was considered consent to participate.

### Consent for publication

Not applicable.

### Availability of data and materials

The survey instrument, and all aggregated data is reported in the manuscript. The database can be made available on request to the authors.

## Additional file

Additional file 1: E-Tables describing respondent demographics, and the details of responses in the 4 different public groups surveyed. **Table E-1**. Demographics of survey respondents. **Table E-2**. Public survey results for questions about “Benefits Arguments” to morally justify animal research. Table **E-3**: Public survey results for questions about “Characteristics of non-human-animals arguments” to morally justify animal research. Table **E-4**. Public survey results for questions about “Human exceptionalism arguments” to morally justify animal research. Table **E-5**. Public survey results for general questions about support for animal research. (PDF 165 kb)

## Abbreviations

AMT: amazon mechanical turk
AR: animal research
NHA: non-human animals
SSI: survey sampling international
