# Supplementary material for: The ethics of animal research: a survey of the public and scientists in North America
Source: BMC Med Ethics. 2016 Mar 29;17:17. doi: 10.1186/s12910-016-0100-x (PMC4812627; doi:10.1186/s12910-016-0100-x)
Supplement: Additional file 1: — E-Tables describing respondent demographics, and the details of responses in the 4 different public groups surveyed. Table E-1. Demographics of survey respondents. Table E-2. Public survey results for questions about “Benefits Arguments” to morally justify animal research. Table E-3: Public survey results for questions about “Characteristics of non-human-animals arguments” to morally justify animal research. Table E-4. Public survey results for questions about “Human exceptionalism arguments” to morally justify animal research. Table E-5. Public survey results for general questions about support for animal research. (PDF 165 kb) [file 12910_2016_100_MOESM1_ESM.pdf]

The ethics of animal research: a survey of the public and scientists in North America

Authors: Ari R Joffe MD[1,2], Meredith Bara BSc[3], Natalie Anton MD[1], Nathan Nobis PhD[4]

Affiliations: [1]University of Alberta, Faculty of Medicine, Department of Pediatrics, Stollery Children's Hospital, Edmonton, Alberta, Canada; [2]University of Alberta, John Dossetor Health Ethics Center; [3]University of Alberta, Faculty of Medicine; [4]Morehouse College, Department of Philosophy, Atlanta, USA.

Corresponding Author: Ari R Joffe MD; 4-546 Edmonton Clinic Health Academy; 11405 87 Avenue; Edmonton, Alberta, Canada; T6G 1C9. Phone: 780 2485435. Email: ari.joffe@albertahealthservices.ca

Journal: BMC Medical Ethics

Additional File 1

Title: E-Tables describing respondent demographics, and the details of responses in the 4 different public groups surveyed.

Description of data:

E-Table 1. Demographics of survey respondents.

E-Table 2. Public survey results for questions about "Benefits Arguments" to morally justify animal research.

E-Table 3: Public survey results for questions about "Characteristics of non-human-animals arguments" to morally justify animal research.

E-Table 4. Public survey results for questions about "Human exceptionalism arguments" to morally justify animal research.

E-Table 5. Public survey results for general questions about support for animal research.

E-Table 1. Demographics of respondents.

Description	Local Festival (n=195)	SSI Survey (n=586)	MTurk Survey (n=439)	Hospital (n=107)	Medical School (n=214)
Age:					
18-24yr	40/193 (21%)	83/585 (14%)	72/439 (16%)	6/107 (6%)	156/214 (73%)
25-34yr	66/193 (34%)	109/585 (19%)	195/439 (44%)	45/107 (42%)	56/214 (26%)
35-44yr	40/193 (21%)	96/585 (16%)	90/439 (21%)	35/107 (33%)	0
45-54yr	23/193 (12%)	110/585 (19%)	48/439 (11%)	13/107 (12%)	1/214 (0.5%)
>54yr	24/193 (12%)	187/585 (32%)	34/439 (8%)	8/107 (8%)	0
Sex					
Male	82/188 (44%)	256/585 (44%)	241/439 (55%)	34/104 (33%)	105/212 (50%)
Education Level					
High school graduate	33/191 (17%)	110/586 (19%)	58/439 (13%)	25/107 (23%)	1/214 (0.5%)
Some College/University	47/191 (25%)	115/586 (20%)	136/439 (31%)	24/107 (22%)	36/214 (17%)
University graduate	105/191 (55%)	361/586 (61%)	243/439 (53%)	49/107 (46%)	176/214 (82%)
None of above	8/191 (4%)	1/586 (0.2%)	3/439 (1%)	9/107 (8%)	0
Work in Medical Field					
No	157/192 (82%)	540/583 (93%)	413/436 (95%)	87/107 (81%)	-
Physician/nurse/student	11/192 (6%)	43/583 (7%)	12/436 (3%)	5/107 (5%)	-
Work in AR					
Have done AR in past	13/186 (7%)	33/584 (6%)	18/436 (4%)	1/104 (1%)	81/214 (38%)
Currently do AR	1/186 (1%)	9/584 (2%)	5/436 (1%)	0	3/214 (1%)
Have never done AR	172/186 (93%)	542/584 (93%)	413/436 (95%)	103/104 (99%)	130/214 (61%)

E-Table 2. Public survey results for questions about "Benefits Arguments" to morally justify animal research.

**Argument (A)/Counterargument (CA)**						
	**Group**	**Is this a good enough reason to justify using animals in medical research?**		**Do any of the following responses make the argument much less convincing?**		**Of those initially convinced: proportion who judged the counterargument as persuasive**
		**Yes**	**No**	**Yes**	**No**	
A1. Animal experimentation benefits humans greatly.						
	Local	91/193 (47%)	102/193 (53%)			
	SSI	293/543 (54%)	250/543 (46%)			
	MTurk	245/429 (57%)	184/429 (43%)			
	Hospital	69/105 (66%)	36/105 (34%)			
	TOTAL	698/1270 (55%)	572/1270 (45%)			
CA: If great human benefits justify using animals in medical research, this should also justify using humans in the same medical research.						
	Local			93/186 (50%)	93/186 (50%)	42/88 (48%)
	SSI			286/544 (53%)	258/544 (47%)	147/292 (50%)
	MTurk			194/428 (45%)	234/428 (55%)	78/245 (32%)
	Hospital			37/102 (36%)	65/102 (64%)	24/67 (36%)
	TOTAL			610/1260 (48%)	650/1260 (52%)	291/692 (42%)
CA: If animals can experience pain and suffering, it remains unclear why we morally may use them in experiments for human benefit.						
	Local			126/190 (66%)	64/190 (34%)	54/89 (61%)
	SSI			355/542 (66%)	187/542 (35%)	167/290 (58%)
	MTurk			263/430 (61%)	167/430 (39%)	101/245 (41%)
	Hospital			48/102 (47%)	54/102 (53%)	26/67 (39%)
	TOTAL			792/1264 (63%)	472/1264 (37%)	348/691 (50%)
A2: Animal experimentation is necessary for human benefit.						
	Local	83/192 (57%)	109/192 (57%)			
	SSI	255/533 (48%)	278/533 (52%)			
	MTurk	217/414 (52%)	197/414 (48%)			
	Hospital	66/107 (62%)	41/107 (38%)			
	TOTAL	621/1246 (50%)	625/1246 (50%)			
CA: More humans would benefit if the money spent on animal experiments was instead devoted to humanitarian aid (for example, in developing countries).[1]						
	Local			108/188 (57%)	80/188 (43%)	40/79 (51%)
	SSI			269/535 (50%)	266/535 (50%)	104/254 (41%)
	MTurk			173/424 (41%)	251/424 (59%)	67/217 (31%)

	Hospital			34/102 (33%)	68/102 (67%)	15/63 (24%)
	TOTAL			584/1249 (47%)	665/1249 (53%)	226/613 (37%)
CA: There are now alternative experimental methods that do not use animals and that allow science to advance.						
	Local			162/186 (87%)	24/186 (13%)	68/80 (85%)
	SSI			453/537 (84%)	84/537 (16%)	200/254 (79%)
	MTurk			358/422 (85%)	64/422 (15%)	171/216 (79%)
	Hospital			76/99 (77%)	23/99 (23%)	43/62 (69%)
	TOTAL			1049/1244 (84%)	195/1244 (16%)	482/612 (79%)
CA: It is unclear why the statement animal experimentation is necessary for human benefits justifies animal experiments, but the statement human experimentation is necessary for human benefits does not justify the same experiments on humans.[1]						
	Local			109/179 (61%)	70/179 (39%)	39/78 (50%)
	SSI			315/534 (59%)	219/534 (41%)	125/253 (49%)
	MTurk			202/424 (48%)	222/424 (52%)	62/217 (29%)
	Hospital			41/101 (41%)	60/101 (59%)	19/64 (30%)
	TOTAL			667/1238 (54%)	571/1238 (46%)	245/612 (40%)
A3: There are no alternatives to animal experimentation.						
	Local	69/191 (36%)	122/191 (64%)			
	SSI	196/521 (38%)	325/521 (62%)			
	MTurk	191/421 (45%)	230/421 (55%)			
	Hospital	51/107 (48%)	56/107 (52%)			
	TOTAL	507/1240 (41%)	733/1240 (59%)			
CA: Researchers have not looked hard enough for alternatives to animal experimentation. For example, since using animals to test drugs has been required by law, researchers may have assumed that there is no other way.						
	Local			126/186 (68%)	60/186 (32%)	44/65 (68%)
	SSI			359/527 (68%)	168/527 (32%)	119/194 (61%)
	MTurk			264/420 (63%)	156/420 (37%)	96/190 (51%)
	Hospital			52/102 (51%)	50/102 (49%)	21/49 (43%)
	TOTAL			801/1235 (65%)	434/1235 (35%)	280/498 (56%)
CA: If more effort was devoted to developing alternative research methods that do not use animals, animal experimentation may not be necessary anymore.						
	Local			151/188 (80%)	37/188 (20%)	50/67 (75%)
	SSI			424/528 (80%)	104/528 (20%)	139/194 (72%)
	MTurk			338/421 (80%)	83/421 (20%)	132/191 (69%)
	Hospital			72/102 (71%)	30/102 (29%)	31/49 (63%)

	TOTAL			985/1239 (79%)	254/1239 (21%)	352/501 (70%)
A4: Humans naturally need to seek knowledge.[1]						
	Local	56/193 (29%)	137/193 (71%)			
	SSI	140/522 (27%)	382/522 (73%)			
	MTurk	63/418 (15%)	355/418 (85%)			
	Hospital	34/107 (32%)	73/107 (68%)			
	TOTAL	293/1240 (24%)	947/1240 (76%)			
CA: This can justify almost anything, including harmful experiments on humans against their will, in order to gain knowledge.[1]						
	Local			98/182 (54%)	84/182 (46%)	27/53 (51%)
	SSI			251/523 (48%)	272/523 (52%)	59/139 (42%)
	MTurk			299/419 (71%)	120/419 (29%)	27/63 (43%)
	Hospital			42/103 (41%)	61/103 (59%)	14/34 (41%)
	TOTAL			690/1227 (56%)	537/1227 (44%)	127/289 (44%)
CA: We have learned a great deal from earthquakes, fires and warfare; but, this does not justify recreating these things in order to gain more knowledge.						
	Local			121/181 (67%)	60/181 (33%)	35/52 (67%)
	SSI			376/529 (71%)	153/529 (29%)	89/140 (64%)
	MTurk			300/420 (71%)	120/420 (29%)	25/63 (40%)
	Hospital			62/101 (61%)	39/101 (39%)	18/32 (56%)
	TOTAL			859/1231 (70%)	371/1231 (30%)	167/287 (58%)

1. Statistically significant difference between the public groups ($p<0.05$ after Bonferroni correction). 2. Clinically significant difference between the public groups (statistically significant, and a clear majority of at least 60% on opposite sides of the yes/no response option): none.

E-Table 3: Public survey results for questions about "Characteristics of non-human-animals arguments" to morally justify animal research.

**Argument (A)/Counterargument (CA)**						
	**Group**	**Is this a good enough reason to justify using animals in medical research?**		**Do any of the following responses make the argument much less convincing?**		**Of those initially convinced: proportion who judged the counterargument as persuasive**
		**Yes**	**No**	**Yes**	**No**	
A1. Animals harm other animals.						
	Local	33/191 (17%)	158/191 (83%)			
	SSI	73/521 (14%)	448/521 (86%)			
	MTurk	32/418 (8%)	386/418 (92%)			
	Hospital	15/107 (14%)	92/107 (86%)			
	TOTAL	153/1237 (12%)	1084/1237 (88%)			
CA: It is unclear why we should take this (we may harm animals) as moral advice from animals, but not take other moral advice from animals (for example, animals rape and kill members of their own species would mean we may rape and kill humans). In other words, animals are not qualified to give moral advice.[1]						
	Local			89/184 (48%)	95/184 (52%)	13/30 (43%)
	SSI			257/525 (49%)	268/525 (51%)	46/72 (64%)
	MTurk			261/415 (63%)	154/415 (37%)	13/31 (42%)
	Hospital			48/103 (47%)	55/103 (53%)	8/15 (53%)
	TOTAL			655/1227 (53%)	572/1227 (47%)	80/148 (54%)
Please tell us whether you agree with this equation: 2 + 2 = 4						
	MTurk	SA/A 407/418 (97%)		U/D/SD 11/418 (3%)		
A2: Animals cannot really feel anything. They are simply living machines.						
	Local	16/190 (8%)	174/190 (92%)			
	SSI	49/523 (9%)	474/523 (91%)			
	MTurk	15/419 (4%)	404/419 (96%)			
	Hospital	4/107 (4%)	103/107 (96%)			
	TOTAL	84/1239 (7%)	1155/1239 (93%)			
CA: This would mean that a pet cat or dog is simply a living machine, without any feelings like happiness, sadness, fear or pain.[1,2]						
	Local			72/188 (38%)	116/188 (62%)	9/15 (60%)
	SSI			185/524 (35%)	339/524 (65%)	38/49 (78%)
	MTurk			277/420 (66%)	143/420 (34%)	11/15 (73%)
	Hospital			40/105 (38%)	65/105 (62%)	2/4 (50%)
	TOTAL			574/1237 (46%)	663/1237 (54%)	60/83 (72%)

A3: Animals are property.[1]						
	Local	34/191 (18%)	157/191 (82%)			
	SSI	74/504 (15%)	430/504 (85%)			
	MTurk	31/415 (8%)	384/415 (93%)			
	Hospital	40/105 (38%)	65/105 (62%)			
	TOTAL	179/1215 (15%)	1036/1215 (85%)			
CA: Since animals can desire things, intentionally act to fulfill those desires, and can understand (even dimly) that it is me that wants something and is trying to get it, they are not simply property.[1]						
	Local			105/186 (57%)	81/186 (44%)	18/33 (55%)
	SSI			304/508 (60%)	204/508 (40%)	42/73 (58%)
	MTurk			299/416 (72%)	117/416 (28%)	12/31 (39%)
	Hospital			51/102 (50%)	51/102 (50%)	12/39 (31%)
	TOTAL			759/1212 (63%)	453/1212 (37%)	84/176 (48%)

1. Statistically significant difference between the public groups (p<0.05 after Bonferroni correction). 2. Clinically significant difference between the public groups (statistically significant, and a clear majority of at least 60% on opposite sides of the yes/no response option).

E-Table 4. Public survey results for questions about "Human exceptionalism arguments" to morally justify animal research.

**Argument (A)/Counterargument (CA)**						
	**Group**	**Is this a good enough reason to justify using animals in medical research?**		**Do any of the following responses make the argument much less convincing?**		**Of those initially convinced: proportion who judged the counterargument as persuasive**
		**Yes**	**No**	**Yes**	**No**	
A1.Humans have more advanced mental abilities than animals, like knowing right from wrong, having empathy, planning for the future, and being able to read and talk.						
	Local	55/193 (29%)	138/193 (72%)			
	SSI	121/518 (23%)	397/518 (77%)			
	MTurk	92/418 (22%)	326/418 (78%)			
	Hospital	28/106 (26%)	78/106 (74%)			
	TOTAL	296/1235 (24%)	939/1235 (76%)			
CA: Not all humans have these abilities. Babies, infants, and severely brain damaged children or adults (for example, with very advanced Alzheimer's) do not have these abilities. Some animals may have more abilities than these humans.						
	Local			109/185 (59%)	76/185 (41%)	28/52 (54%)
	SSI			322/519 (62%)	197/519 (38%)	69/121 (57%)
	MTurk			239/418 (57%)	179/418 (43%)	31/92 (34%)
	Hospital			62/104 (60%)	42/104 (40%)	14/28 (50%)
	TOTAL			732/1226 (60%)	494/1226 (40%)	142/293 (48%)
CA: This means having superior abilities [humans] justifies actively harming those with inferior abilities [animals]. It is unclear why, if animals can experience pain and suffering, having lower mental abilities makes it acceptable to use them in experiments. For example, sometimes humans with superior abilities [adults] have many obligations to those with inferior abilities [children].[1]						
	Local			100/184 (54%)	84/184 (46%)	33/51 (65%)
	SSI			216/516 (42%)	300/516 (58%)	63/119 (53%)
	MTurk			271/418 (65%)	147/418 (35%)	39/92 (42%)
	Hospital			47/99 (48%)	52/99 (53%)	12/27 (44%)
	TOTAL			634/1217 (52%)	583/1217 (48%)	147/289 (51%)
A2: Humans are a special kind or group. We care more about this kind, and have more obligations to this kind.						
	Local	51/193 (26%)	142/193 (74%)			
	SSI	130/512 (25%)	382/512 (75%)			
	MTurk	133/412 (32%)	279/412 (68%)			
	Hospital	37/106 (35%)	69/106 (65%)			

	TOTAL	351/1223 (29%)	872/1223 (71%)			
CA: Imagine there is a more advanced species than humans. This would mean that they are justified in using humans in experiments, because they care more about their own kind.[1]						
	Local			100/186 (54%)	86/186 (46%)	28/49 (57%)
	SSI			229/511 (45%)	282/511 (55%)	70/130 (54%)
	MTurk			261/414 (63%)	153/414 (37%)	63/131 (48%)
	Hospital			44/102 (43%)	58/102 (57%)	19/35 (54%)
	TOTAL			634/1213 (52%)	579/1213 (48%)	180/345 (52%)
CA: Maybe humans are of the kind 'able to experience suffering and pleasure' (sentient being). If so, our kind includes animals.						
	Local			112/186 (60%)	74/186 (40%)	26/49 (53%)
	SSI			271/512 (53%)	241/512 (47%)	64/130 (49%)
	MTurk			218/416 (52%)	198/416 (48%)	38/133 (29%)
	Hospital			48/103 (47%)	55/103 (53%)	10/37 (27%)
	TOTAL			649/1217 (53%)	568/1217 (47%)	138/349 (40%)
CA: Maybe humans are of the kind 'able to have experiences, memories, and preferences' (subject of a life). If so, our kind includes animals.[1]						
	Local			126/185 (68%)	59/185 (32%)	29/49 (59%)
	SSI			292/509 (57%)	217/509 (43%)	70/130 (54%)
	MTurk			207/415 (50%)	208/415 (50%)	33/133 (25%)
	Hospital			48/104 (46%)	56/104 (54%)	9/36 (25%)
	TOTAL			673/1213 (55%)	540/1213 (45%)	141/348 (41%)
CA: It is unclear why caring more about someone justifies harming those we care less about. For example, in the past this argument was used to justify prejudice (for example, slavery) against those we cared less about, who were considered not of our own kind.[1]						
	Local			108/183 (59%)	75/183 (41%)	31/49 (63%)
	SSI			262/509 (52%)	247/509 (49%)	67/130 (52%)
	MTurk			280/416 (67%)	136/416 (33%)	63/133 (47%)
	Hospital			50/100 (50%)	50/100 (50%)	15/34 (44%)
	TOTAL			700/1208 (58%)	508/1208 (42%)	176/346 (51%)
A3: We have moral duties only to those who can agree to the same duties. This is like a contract between people in society. Since animals cannot enter into this contract with humans, we do not have moral duties to animals.						
	Local	37/193 (19%)	156/193 (81%)			
	SSI	100/511 (20%)	411/511 (80%)			
	MTurk	64/414 (16%)	350/414 (85%)			
	Hospital	17/105 (16%)	88/105 (84%)			
	TOTAL	218/1223 (18%)	1005/1223 (82%)			

CA: This would mean we have no direct moral duties to humans who cannot enter into this contract. For example, babies, and severely brain-damaged people.[1,2]						
	Local			98/184 (53%)	86/184 (47%)	14/34 (41%)
	SSI			212/512 (41%)	300/512 (59%)	50/100 (50%)
	MTurk			277/416 (67%)	139/416 (33%)	22/64 (34%)
	Hospital			40/104 (39%)	64/104 (62%)	8/17 (47%)
	TOTAL			577/1216 (47%)	589/1216 (48%)	94/215 (44%)
A4: Evolution, and our nature, dictates that we must make sure we survive as a species.						
	Local	69/187 (37%)	118/187 (63%)			
	SSI	158/507 (31%)	349/507 (69%)			
	MTurk	150/414 (36%)	264/414 (64%)			
	Hospital	41/106 (39%)	65/106 (61%)			
	TOTAL	418/1214 (34%)	796/1214 (66%)			
CA: It is unclear why what we evolved to do [survive at all costs] is what we morally should do. In other words, evolution does not take moral considerations into account.						
	Local			116/183 (63%)	67/183 (37%)	34/65 (52%)
	SSI			277/506 (55%)	229/506 (45%)	96/156 (62%)
	MTurk			232/416 (56%)	184/416 (44%)	57/150 (38%)
	Hospital			41/103 (40%)	62/103 (60%)	14/41 (34%)
	TOTAL			666/1208 (55%)	542/1208 (45%)	201/412 (49%)
CA: Research is unlikely to save our species; it is for the benefit of some humans, not the whole species (which is what evolution is about).						
	Local			91/181 (50%)	90/181 (50%)	31/64 (48%)
	SSI			251/508 (49%)	257/508 (51%)	76/158 (48%)
	MTurk			172/412 (42%)	240/412 (58%)	32/148 (22%)
	Hospital			36/102 (35%)	66/102 (65%)	14/40 (35%)
	TOTAL			550/1203 (46%)	653/1203 (54%)	153/410 (37%)
To show you have read the instructions, please check "none of these" as your answer to this question						
	MTurk	None of these 413/415 (99.5%)		SA/A/U/D 2/415 (0.4%)		
A5: We must sacrifice one (animals) in order to save another (humans). This is like being in a lifeboat on the ocean where we must throw one overboard or the lifeboat will sink.[1]						
	Local	68/187 (36%)	119/187 (64%)			
	SSI	174/507 (34%)	333/507 (66%)			
	MTurk	179/414 (43%)	235/414 (57%)			
	Hospital	17/104 (16%)	87/104 (84%)			

	TOTAL	438/1212 (36%)	774/1212 (64%)			
CA: Most people would throw a dog overboard to save humans in the lifeboat; but, this does not mean that the dog can be used in experiments. For example, some might throw an elderly man overboard to save their children in the lifeboat; but, this does not mean elderly men can be used for experiments.						
	Local			101/182 (56%)	81/182 (45%)	34/67 (51%)
	SSI			269/506 (53%)	237/506 (47%)	87/172 (51%)
	MTurk			228/416 (55%)	188/416 (45%)	54/179 (30%)
	Hospital			52/102 (51%)	50/102 (49%)	4/17 (24%)
	TOTAL			650/1206 (54%)	556/1206 (46%)	179/435 (41%)

1. Statistically significant difference between the public groups ($p<0.05$ after Bonferroni correction). 2. Clinically significant difference between the public groups (statistically significant, and a clear majority of at least 60% on opposite sides of the yes/no response option).

E-Table 5. Public survey responses to general questions about support for animal research.

**Question**	**Group**	**Yes**	**No**	**I have never thought about whether AR should be supported**
In order to achieve human benefits, research that results in harm to animals (such as pain, suffering and early death) should be supported.[1]	Local Festival	73/190 (38%)	93/190 (49%)	24/190 (13%)
	SSI	239/576 (42%)	236/576 (41%)	101/576 (18%)
	MTurk	210/435 (48%)	188/435 (43%)	37/435 (9%)
	Hospital	47/102 (46%)	33/102 (32%)	22/102 (22%)
	TOTAL	569/1303 (44%)	550/1303 (42%)	184/1303 (14%)
Considering all the arguments and responses in this survey, we want to ask you again.[1]	Local Festival	81/189 (43%)	108/189 (57%)	-
	SSI	175/504 (35%)	329/504 (65%)	-
	MTurk	186/414 (45%)	228/414 (55%)	-
	Hospital	60/106 (57%)	46/106 (43%)	-
	TOTAL	502/1213 (41%)	711/1213 (59%)	-
Of those who originally said "yes" or "have never thought about whether to support AR".	Local Festival		22/92 (24%)	-
	SSI		140/298 (47%)	-
	MTurk		54/234 (23%)	-
	Hospital		13/68 (19%)	-
	TOTAL		229/692 (33%)	-
**What is it about vulnerable humans (for example babies, severely brain damaged people, people with very advanced Alzheimers) that makes it wrong to use them in experiments?[1]**	**These vulnerable human are able to experience things like pleasure, joy, happiness, sadness pain, and suffering**	**These humans are vulnerable to physical and psychological harm; using them in experiments is harmful for them**	**We care about them**	**They are still human**
Local Festival	42/163 (26%)	25/163 (15%)	11/163 (7%)	85/163 (52%)
SSI	108/507 (21%)	94/507 (19%)	73/507 (14%)	232/507 (46%)
MTurk	97/415 (23%)	78/415 (19%)	28/415 (7%)	212/415 (51%)
Hospital	21/103 (20%)	11/103 (11%)	13/103 (13%)	58/103 (56%)
TOTAL	268/1188 (23%)	208/1188 (18%)	125/1188 (11%)	587/1188 (49%)

1. Statistically significant difference between the public groups ($p<0.05$ after Bonferroni correction). 2. Clinically significant difference between the public groups (statistically significant, and a clear majority of at least 60% on opposite sides of the response options): none.
